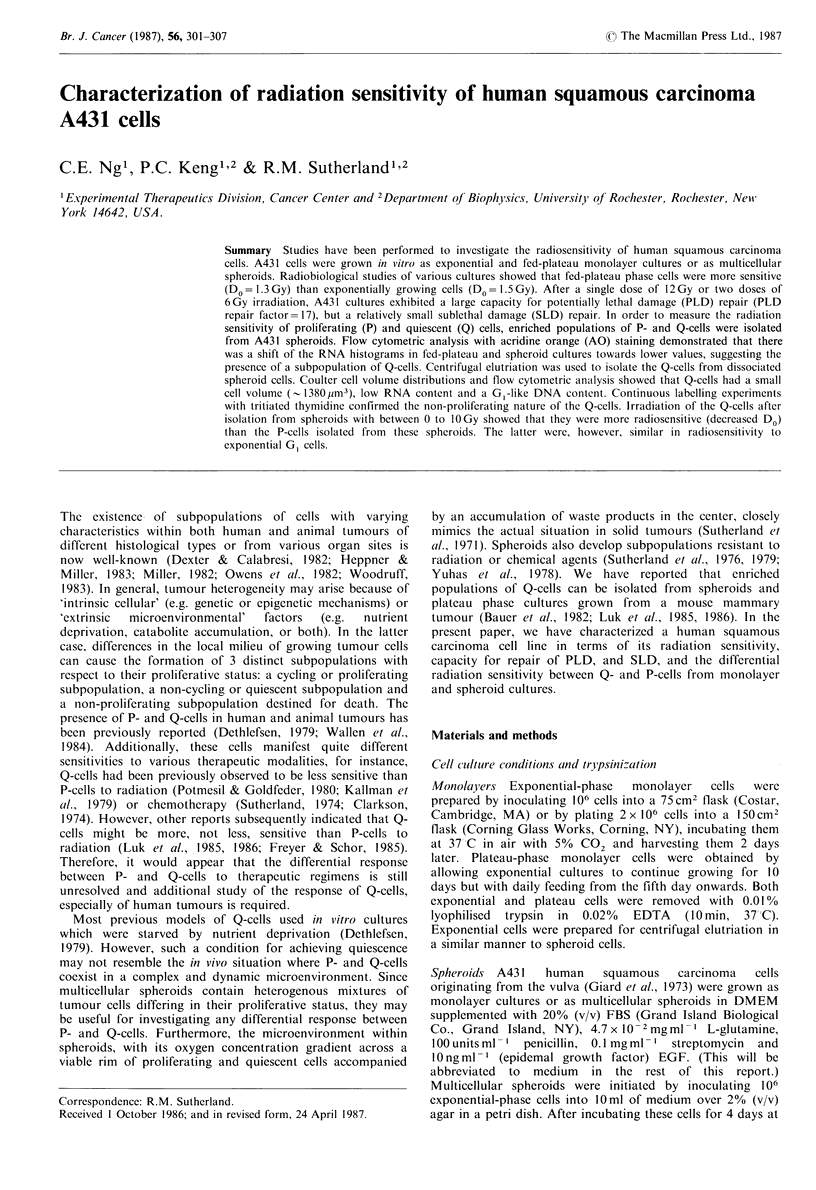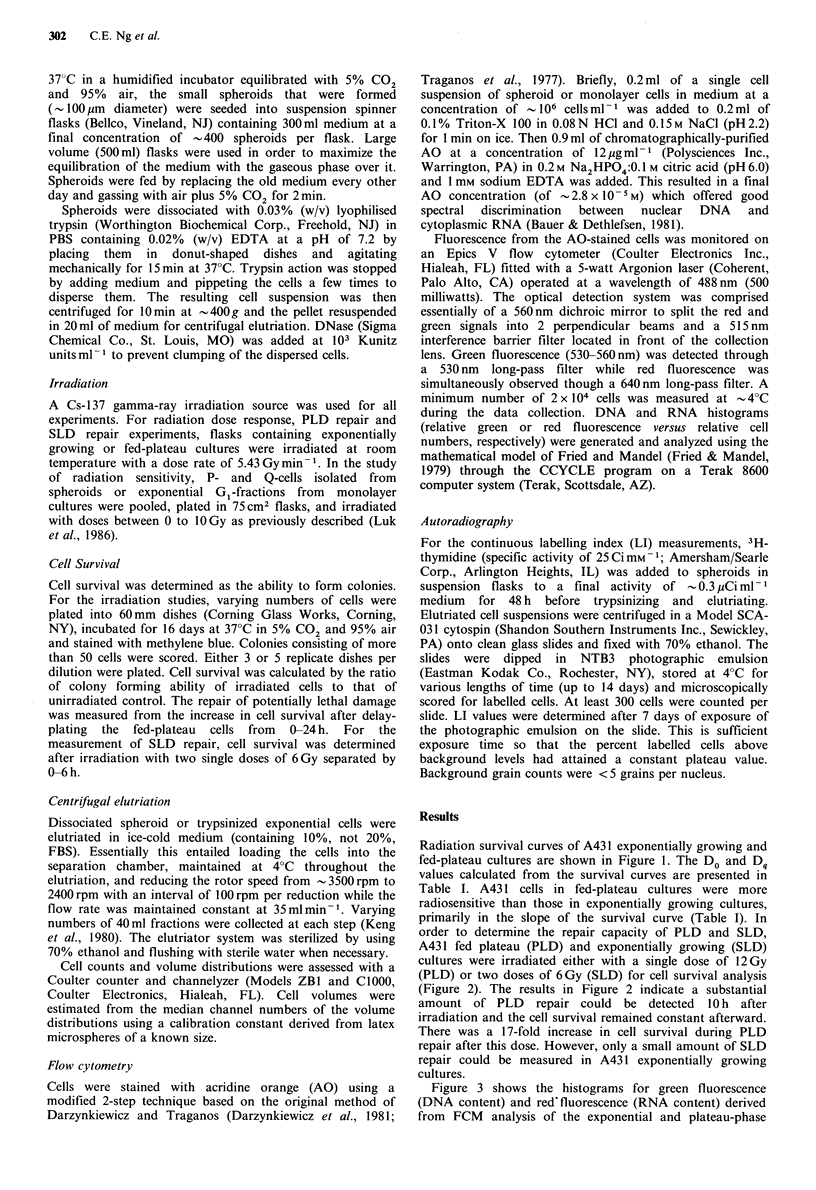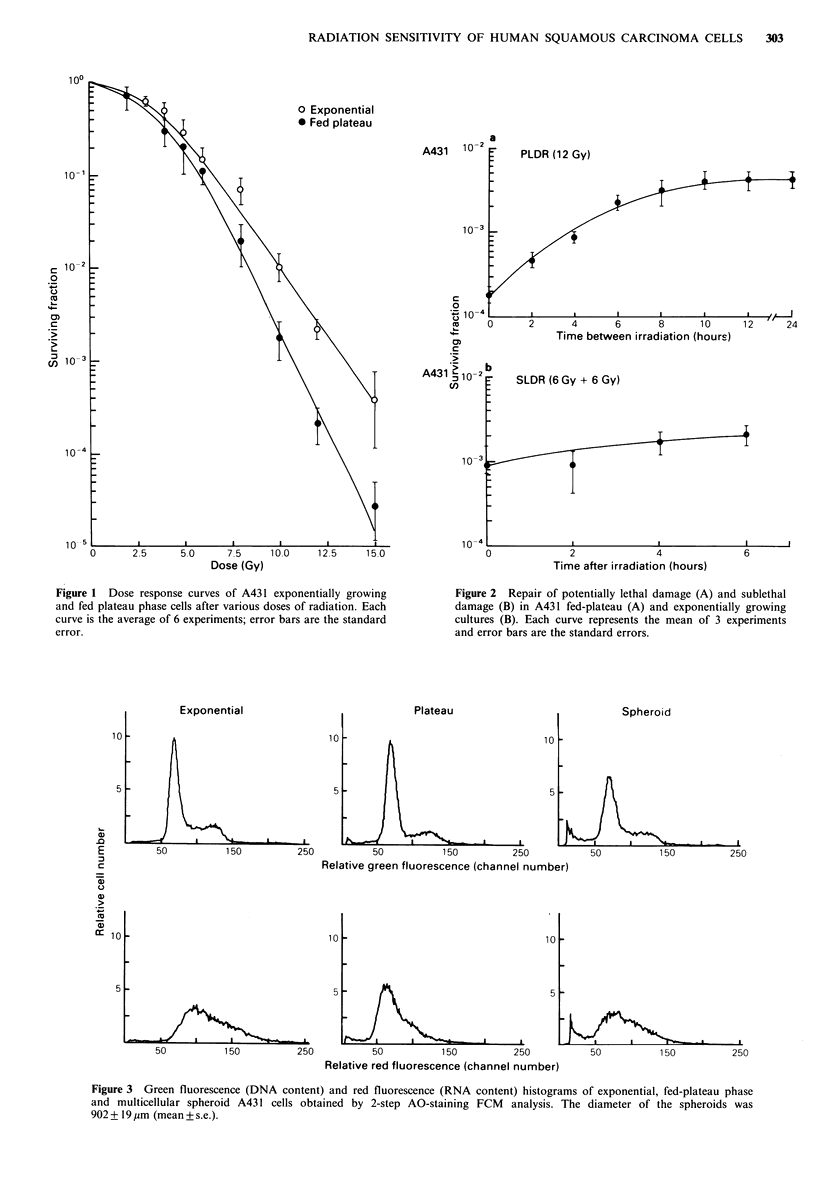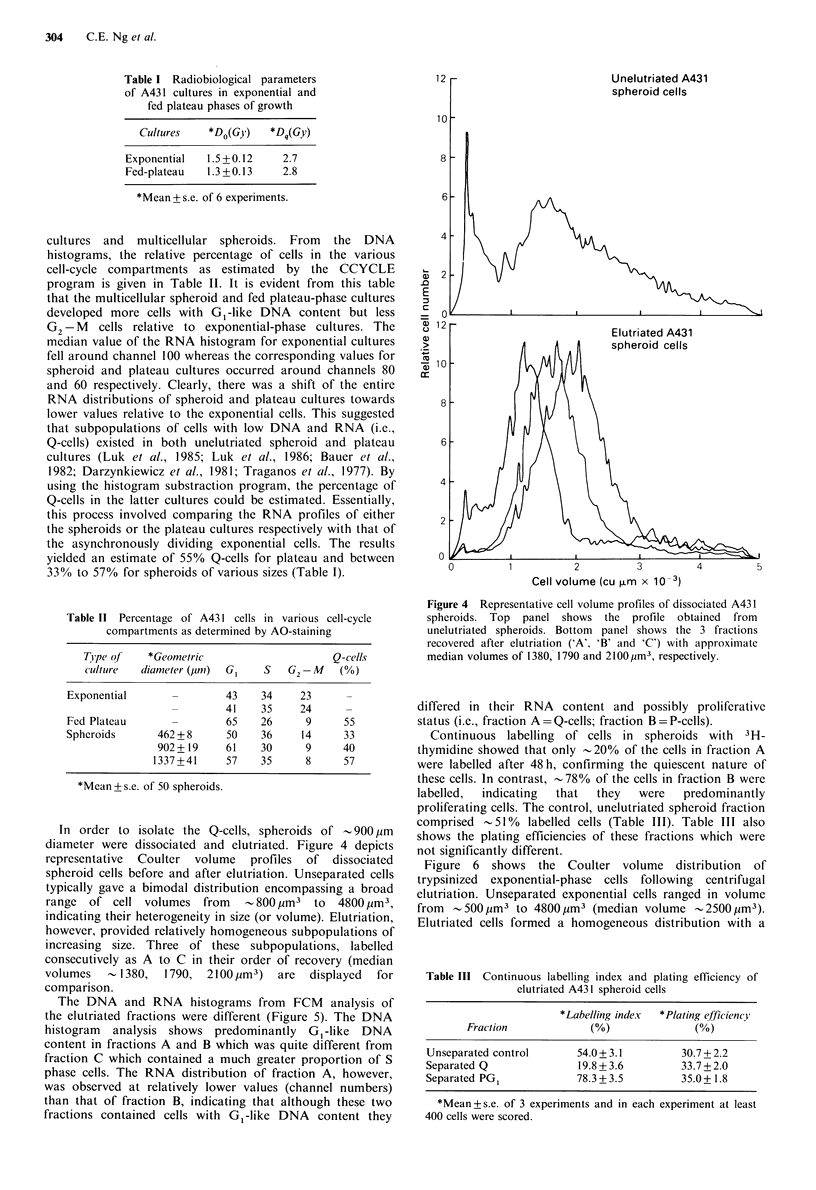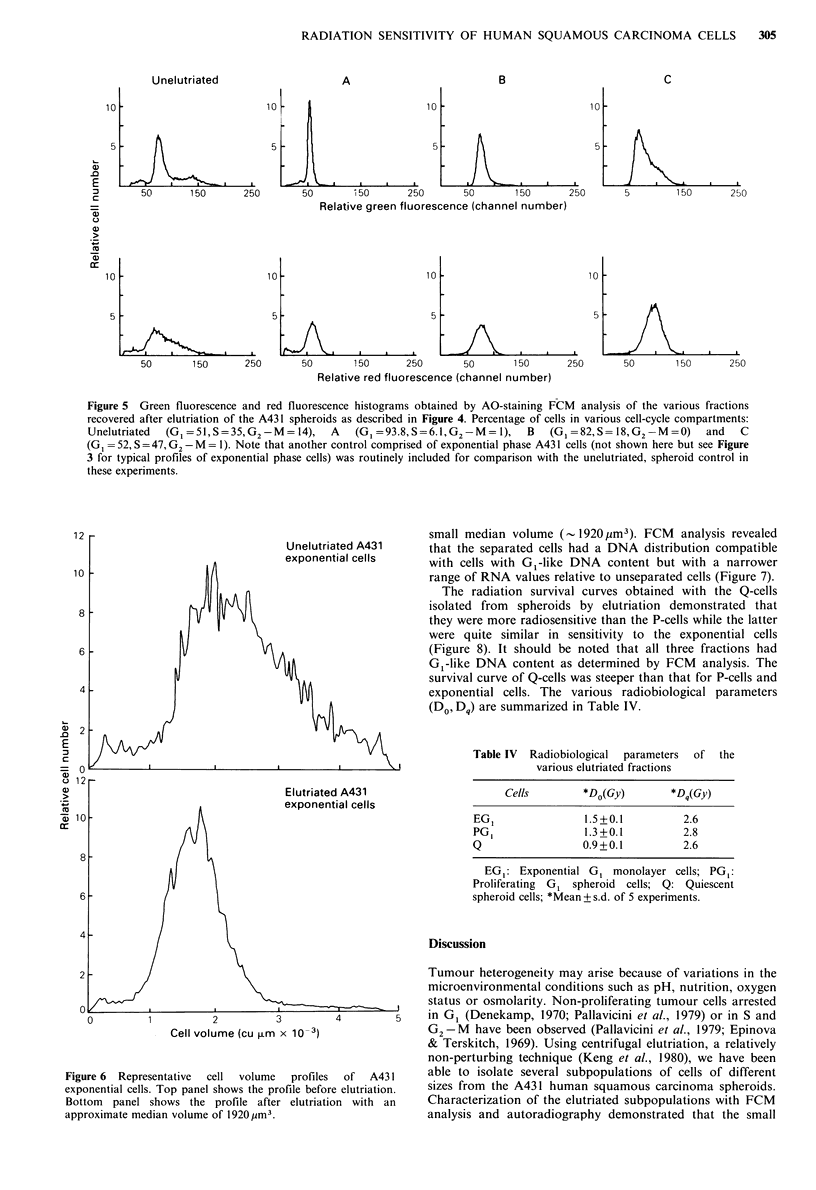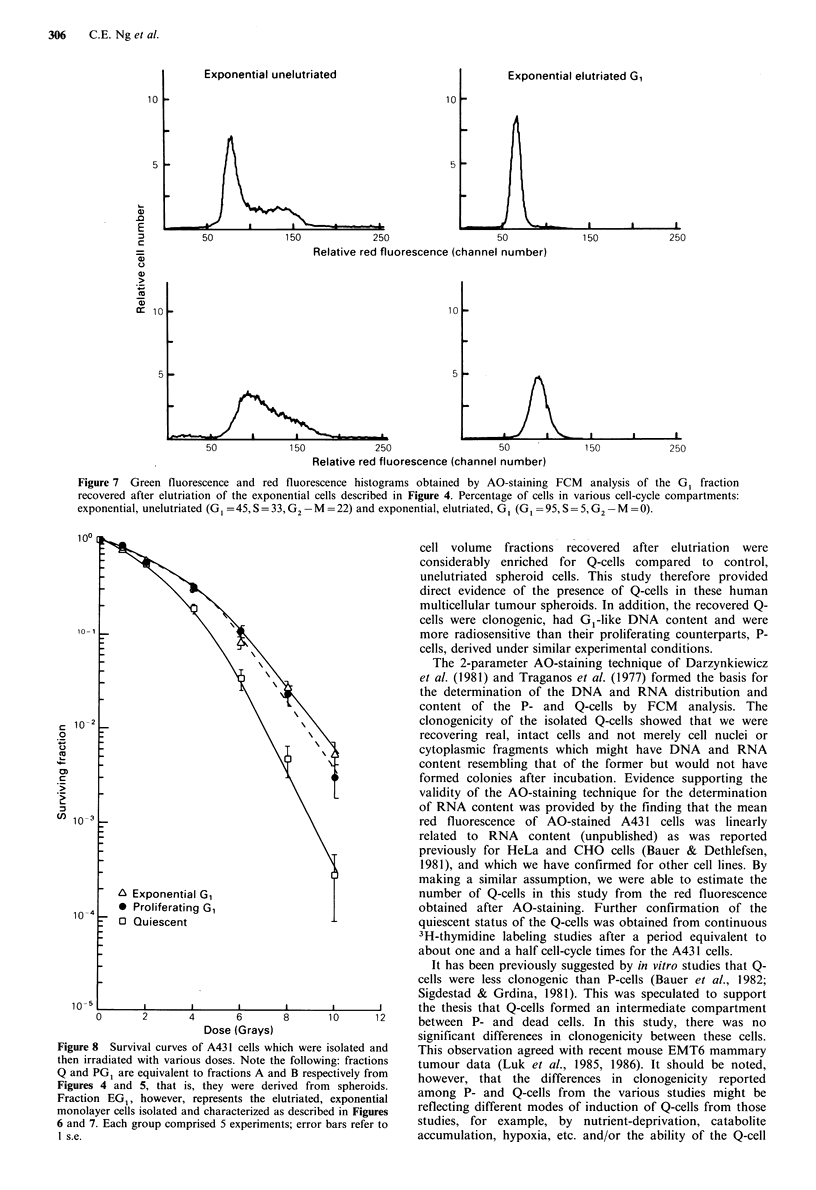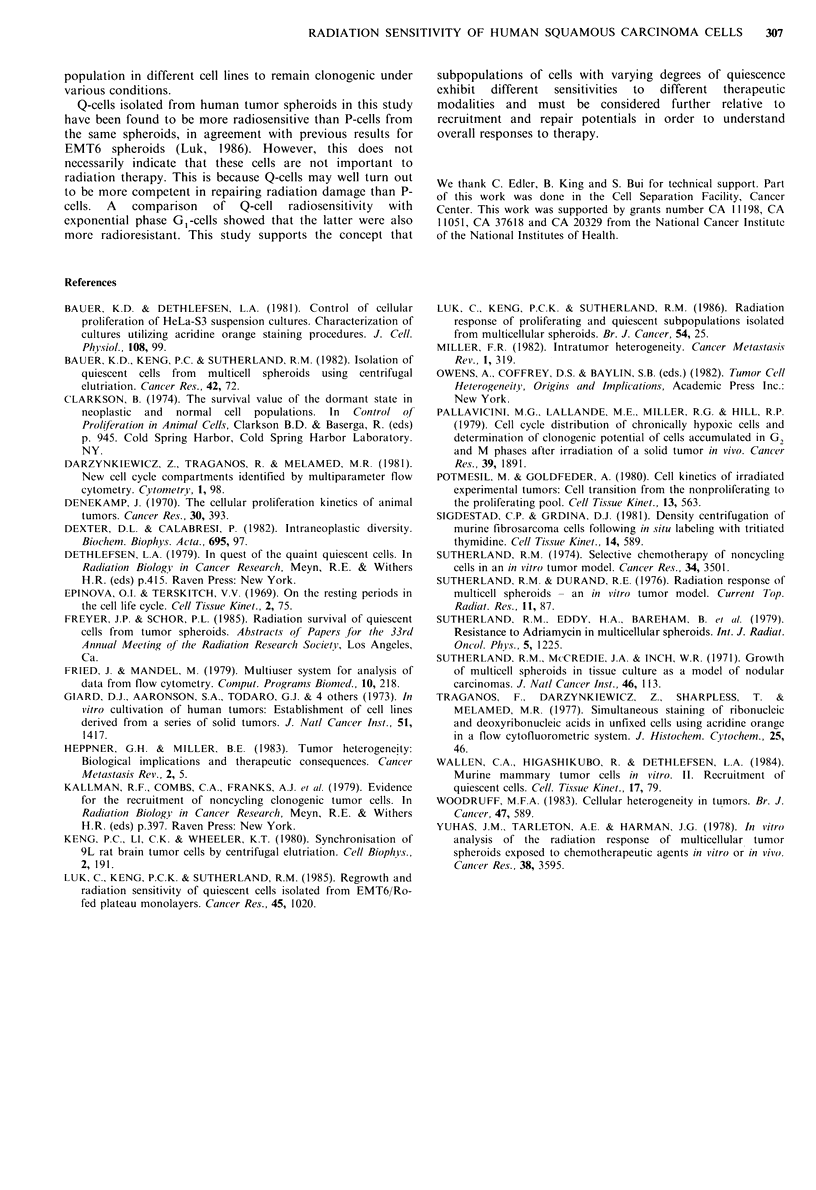# Characterization of radiation sensitivity of human squamous carcinoma A431 cells.

**DOI:** 10.1038/bjc.1987.193

**Published:** 1987-09

**Authors:** C. E. Ng, P. C. Keng, R. M. Sutherland

**Affiliations:** Cancer Center, University of Rochester, New York 14642.

## Abstract

Studies have been performed to investigate the radiosensitivity of human squamous carcinoma cells. A431 cells were grown in vitro as exponential and fed-plateau monolayer cultures or as multicellular spheroids. Radiobiological studies of various cultures showed that fed-plateau phase cells were more sensitive (D0 = 1.3 Gy) than exponentially growing cells (D0 = 1.5 Gy). After a single dose of 12 Gy or two doses of 6 Gy irradiation, A431 cultures exhibited a large capacity for potentially lethal damage (PLD) repair (PLD repair factor = 17), but a relatively small sublethal damage (SLD) repair. In order to measure the radiation sensitivity of proliferating (P) and quiescent (Q) cells, enriched populations of P- and Q-cells were isolated from A431 spheroids. Flow cytometric analysis with acridine orange (AO) staining demonstrated that there was a shift of the RNA histograms in fed-plateau and spheroid cultures towards lower values, suggesting the presence of a subpopulation of Q-cells. Centrifugal elutriation was used to isolate the Q-cells from dissociated spheroid cells. Coulter cell volume distributions and flow cytometric analysis showed that Q-cells had a small cell volume (approximately 1380 microns3), low RNA content and a G1-like DNA content. Continuous labelling experiments with tritiated thymidine confirmed the non-proliferating nature of the Q-cells. Irradiation of the Q-cells after isolation from spheroids with between 0 to 10 Gy showed that they were more radiosensitive (decreased D0) than the P-cells isolated from these spheroids. The latter were, however, similar in radiosensitivity to exponential G1 cells.


 © The Macmillan Press Ltd., 1987

### Characterization of radiation sensitivity of human squamous carcinoma A431 cells

C.E. Ng[1], P.C. Keng[1,2] & R.M. Sutherland[1,2]

[1]*Experimental Therapeutics Division, Cancer Center and* [2]*Department of Biophysics, University of Rochester, Rochester, New York 14642, USA.*

**Summary** Studies have been performed to investigate the radiosensitivity of human squamous carcinoma cells. A431 cells were grown *in vitro* as exponential and fed-plateau monolayer cultures or as multicellular spheroids. Radiobiological studies of various cultures showed that fed-plateau phase cells were more sensitive ($D_0 = 1.3$ Gy) than exponentially growing cells ($D_0 = 1.5$ Gy). After a single dose of 12 Gy or two doses of 6 Gy irradiation, A431 cultures exhibited a large capacity for potentially lethal damage (PLD) repair (PLD repair factor = 17), but a relatively small sublethal damage (SLD) repair. In order to measure the radiation sensitivity of proliferating (P) and quiescent (Q) cells, enriched populations of P- and Q-cells were isolated from A431 spheroids. Flow cytometric analysis with acridine orange (AO) staining demonstrated that there was a shift of the RNA histograms in fed-plateau and spheroid cultures towards lower values, suggesting the presence of a subpopulation of Q-cells. Centrifugal elutriation was used to isolate the Q-cells from dissociated spheroid cells. Coulter cell volume distributions and flow cytometric analysis showed that Q-cells had a small cell volume ($\sim 1380\,\mu m^3$), low RNA content and a $G_1$-like DNA content. Continuous labelling experiments with tritiated thymidine confirmed the non-proliferating nature of the Q-cells. Irradiation of the Q-cells after isolation from spheroids with between 0 to 10 Gy showed that they were more radiosensitive (decreased $D_0$) than the P-cells isolated from these spheroids. The latter were, however, similar in radiosensitivity to exponential $G_1$ cells.

The existence of subpopulations of cells with varying characteristics within both human and animal tumours of different histological types or from various organ sites is now well-known (Dexter & Calabresi, 1982; Heppner & Miller, 1983; Miller, 1982; Owens *et al.*, 1982; Woodruff, 1983). In general, tumour heterogeneity may arise because of 'intrinsic cellular' (e.g. genetic or epigenetic mechanisms) or 'extrinsic microenvironmental' factors (e.g. nutrient deprivation, catabolite accumulation, or both). In the latter case, differences in the local milieu of growing tumour cells can cause the formation of 3 distinct subpopulations with respect to their proliferative status: a cycling or proliferating subpopulation, a non-cycling or quiescent subpopulation and a non-proliferating subpopulation destined for death. The presence of P- and Q-cells in human and animal tumours has been previously reported (Dethlefsen, 1979; Wallen *et al.*, 1984). Additionally, these cells manifest quite different sensitivities to various therapeutic modalities, for instance, Q-cells had been previously observed to be less sensitive than P-cells to radiation (Potmesil & Goldfeder, 1980; Kallman *et al.*, 1979) or chemotherapy (Sutherland, 1974; Clarkson, 1974). However, other reports subsequently indicated that Q-cells might be more, not less, sensitive than P-cells to radiation (Luk *et al.*, 1985, 1986; Freyer & Schor, 1985). Therefore, it would appear that the differential response between P- and Q-cells to therapeutic regimens is still unresolved and additional study of the response of Q-cells, especially of human tumours is required.

Most previous models of Q-cells used *in vitro* cultures which were starved by nutrient deprivation (Dethlefsen, 1979). However, such a condition for achieving quiescence may not resemble the *in vivo* situation where P- and Q-cells coexist in a complex and dynamic microenvironment. Since multicellular spheroids contain heterogenous mixtures of tumour cells differing in their proliferative status, they may be useful for investigating any differential response between P- and Q-cells. Furthermore, the microenvironment within spheroids, with its oxygen concentration gradient across a viable rim of proliferating and quiescent cells accompanied by an accumulation of waste products in the center, closely mimics the actual situation in solid tumours (Sutherland *et al.*, 1971). Spheroids also develop subpopulations resistant to radiation or chemical agents (Sutherland *et al.*, 1976, 1979; Yuhas *et al.*, 1978). We have reported that enriched populations of Q-cells can be isolated from spheroids and plateau phase cultures grown from a mouse mammary tumour (Bauer *et al.*, 1982; Luk *et al.*, 1985, 1986). In the present paper, we have characterized a human squamous carcinoma cell line in terms of its radiation sensitivity, capacity for repair of PLD, and SLD, and the differential radiation sensitivity between Q- and P-cells from monolayer and spheroid cultures.

### Materials and methods

### *Cell culture conditions and trypsinization*

*Monolayers* Exponential-phase monolayer cells were prepared by inoculating $10^6$ cells into a 75 $cm^2$ flask (Costar, Cambridge, MA) or by plating $2 \times 10^6$ cells into a 150 $cm^2$ flask (Corning Glass Works, Corning, NY), incubating them at 37°C in air with 5% $CO_2$ and harvesting them 2 days later. Plateau-phase monolayer cells were obtained by allowing exponential cultures to continue growing for 10 days but with daily feeding from the fifth day onwards. Both exponential and plateau cells were removed with 0.01% lyophilised trypsin in 0.02% EDTA (10 min, 37°C). Exponential cells were prepared for centrifugal elutriation in a similar manner to spheroid cells.

*Spheroids* A431 human squamous carcinoma cells originating from the vulva (Giard *et al.*, 1973) were grown as monolayer cultures or as multicellular spheroids in DMEM supplemented with 20% (v/v) FBS (Grand Island Biological Co., Grand Island, NY), $4.7 \times 10^{-2}$ mg $ml^{-1}$ L-glutamine, 100 units $ml^{-1}$ penicillin, 0.1 mg $ml^{-1}$ streptomycin and 10 ng $ml^{-1}$ (epidemal growth factor) EGF. (This will be abbreviated to medium in the rest of this report.) Multicellular spheroids were initiated by inoculating $10^6$ exponential-phase cells into 10 ml of medium over 2% (v/v) agar in a petri dish. After incubating these cells for 4 days at

Correspondence: R.M. Sutherland.
Received 1 October 1986; and in revised form, 24 April 1987.

37°C in a humidified incubator equilibrated with 5% $CO_2$ and 95% air, the small spheroids that were formed (~100 μm diameter) were seeded into suspension spinner flasks (Bellco, Vineland, NJ) containing 300 ml medium at a final concentration of ~400 spheroids per flask. Large volume (500 ml) flasks were used in order to maximize the equilibration of the medium with the gaseous phase over it. Spheroids were fed by replacing the old medium every other day and gassing with air plus 5% $CO_2$ for 2 min.

Spheroids were dissociated with 0.03% (w/v) lyophilised trypsin (Worthington Biochemical Corp., Freehold, NJ) in PBS containing 0.02% (w/v) EDTA at a pH of 7.2 by placing them in donut-shaped dishes and agitating mechanically for 15 min at 37°C. Trypsin action was stopped by adding medium and pippeting the cells a few times to disperse them. The resulting cell suspension was then centrifuged for 10 min at ~400 *g* and the pellet resuspended in 20 ml of medium for centrifugal elutriation. DNase (Sigma Chemical Co., St. Louis, MO) was added at $10^3$ Kunitz units $ml^{-1}$ to prevent clumping of the dispersed cells.

*Irradiation*

A Cs-137 gamma-ray irradiation source was used for all experiments. For radiation dose response, PLD repair and SLD repair experiments, flasks containing exponentially growing or fed-plateau cultures were irradiated at room temperature with a dose rate of 5.43 Gy $min^{-1}$. In the study of radiation sensitivity, P- and Q-cells isolated from spheroids or exponential $G_1$-fractions from monolayer cultures were pooled, plated in 75 $cm^2$ flasks, and irradiated with doses between 0 to 10 Gy as previously described (Luk *et al.*, 1986).

*Cell Survival*

Cell survival was determined as the ability to form colonies. For the irradiation studies, varying numbers of cells were plated into 60 mm dishes (Corning Glass Works, Corning, NY), incubated for 16 days at 37°C in 5% $CO_2$ and 95% air and stained with methylene blue. Colonies consisting of more than 50 cells were scored. Either 3 or 5 replicate dishes per dilution were plated. Cell survival was calculated by the ratio of colony forming ability of irradiated cells to that of unirradiated control. The repair of potentially lethal damage was measured from the increase in cell survival after delay-plating the fed-plateau cells from 0–24 h. For the measurement of SLD repair, cell survival was determined after irradiation with two single doses of 6 Gy separated by 0–6 h.

*Centrifugal elutriation*

Dissociated spheroid or trypsinized exponential cells were elutriated in ice-cold medium (containing 10%, not 20%, FBS). Essentially this entailed loading the cells into the separation chamber, maintained at 4°C throughout the elutriation, and reducing the rotor speed from ~3500 rpm to 2400 rpm with an interval of 100 rpm per reduction while the flow rate was maintained constant at 35 ml $min^{-1}$. Varying numbers of 40 ml fractions were collected at each step (Keng *et al.*, 1980). The elutriator system was sterilized by using 70% ethanol and flushing with sterile water when necessary.

Cell counts and volume distributions were assessed with a Coulter counter and channelyzer (Models ZB1 and C1000, Coulter Electronics, Hialeah, FL). Cell volumes were estimated from the median channel numbers of the volume distributions using a calibration constant derived from latex microspheres of a known size.

*Flow cytometry*

Cells were stained with acridine orange (AO) using a modified 2-step technique based on the original method of Darzynkiewicz and Traganos (Darzynkiewicz *et al.*, 1981; Traganos *et al.*, 1977). Briefly, 0.2 ml of a single cell suspension of spheroid or monolayer cells in medium at a concentration of ~$10^6$ cells $ml^{-1}$ was added to 0.2 ml of 0.1% Triton-X 100 in 0.08 N HCl and 0.15 M NaCl (pH 2.2) for 1 min on ice. Then 0.9 ml of chromatographically-purified AO at a concentration of 12 μg $ml^{-1}$ (Polysciences Inc., Warrington, PA) in 0.2 M $Na_2HPO_4$:0.1 M citric acid (pH 6.0) and 1 mM sodium EDTA was added. This resulted in a final AO concentration (of ~$2.8 \times 10^{-5}$ M) which offered good spectral discrimination between nuclear DNA and cytoplasmic RNA (Bauer & Dethlefsen, 1981).

Fluorescence from the AO-stained cells was monitored on an Epics V flow cytometer (Coulter Electronics Inc., Hialeah, FL) fitted with a 5-watt Argonion laser (Coherent, Palo Alto, CA) operated at a wavelength of 488 nm (500 milliwatts). The optical detection system was comprised essentially of a 560 nm dichroic mirror to split the red and green signals into 2 perpendicular beams and a 515 nm interference barrier filter located in front of the collection lens. Green fluorescence (530–560 nm) was detected through a 530 nm long-pass filter while red fluorescence was simultaneously observed though a 640 nm long-pass filter. A minimum number of $2 \times 10^4$ cells was measured at ~4°C during the data collection. DNA and RNA histograms (relative green or red fluorescence *versus* relative cell numbers, respectively) were generated and analyzed using the mathematical model of Fried and Mandel (Fried & Mandel, 1979) through the CCYCLE program on a Terak 8600 computer system (Terak, Scottsdale, AZ).

*Autoradiography*

For the continuous labelling index (LI) measurements, $^3H$-thymidine (specific activity of 25 Ci $mM^{-1}$; Amersham/Searle Corp., Arlington Heights, IL) was added to spheroids in suspension flasks to a final activity of ~0.3 μCi $ml^{-1}$ medium for 48 h before trypsinizing and elutriating. Elutriated cell suspensions were centrifuged in a Model SCA-031 cytospin (Shandon Southern Instruments Inc., Sewickley, PA) onto clean glass slides and fixed with 70% ethanol. The slides were dipped in NTB3 photographic emulsion (Eastman Kodak Co., Rochester, NY), stored at 4°C for various lengths of time (up to 14 days) and microscopically scored for labelled cells. At least 300 cells were counted per slide. LI values were determined after 7 days of exposure of the photographic emulsion on the slide. This is sufficient exposure time so that the percent labelled cells above background levels had attained a constant plateau value. Background grain counts were <5 grains per nucleus.

### Results

Radiation survival curves of A431 exponentially growing and fed-plateau cultures are shown in Figure 1. The $D_0$ and $D_q$ values calculated from the survival curves are presented in Table I. A431 cells in fed-plateau cultures were more radiosensitive than those in exponentially growing cultures, primarily in the slope of the survival curve (Table I). In order to determine the repair capacity of PLD and SLD, A431 fed plateau (PLD) and exponentially growing (SLD) cultures were irradiated either with a single dose of 12 Gy (PLD) or two doses of 6 Gy (SLD) for cell survival analysis (Figure 2). The results in Figure 2 indicate a substantial amount of PLD repair could be detected 10 h after irradiation and the cell survival remained constant afterward. There was a 17-fold increase in cell survival during PLD repair after this dose. However, only a small amount of SLD repair could be measured in A431 exponentially growing cultures.

Figure 3 shows the histograms for green fluorescence (DNA content) and red fluorescence (RNA content) derived from FCM analysis of the exponential and plateau-phase

Figure 1 Dose response curves of A431 exponentially growing and fed plateau phase cells after various doses of radiation. Each curve is the average of 6 experiments; error bars are the standard error.

Figure 2 Repair of potentially lethal damage (A) and sublethal damage (B) in A431 fed-plateau (A) and exponentially growing cultures (B). Each curve represents the mean of 3 experiments and error bars are the standard errors.

Figure 3 Green fluorescence (DNA content) and red fluorescence (RNA content) histograms of exponential, fed-plateau phase and multicellular spheroid A431 cells obtained by 2-step AO-staining FCM analysis. The diameter of the spheroids was $902 \pm 19\,\mu m$ (mean $\pm$ s.e.).

**Table I** Radiobiological parameters of A431 cultures in exponential and fed plateau phases of growth

| *Cultures* | $*D_0(Gy)$ | $*D_q(Gy)$ |
|---|---|---|
| Exponential | 1.5±0.12 | 2.7 |
| Fed-plateau | 1.3±0.13 | 2.8 |

*Mean±s.e. of 6 experiments.

cultures and multicellular spheroids. From the DNA histograms, the relative percentage of cells in the various cell-cycle compartments as estimated by the CCYCLE program is given in Table II. It is evident from this table that the multicellular spheroid and fed plateau-phase cultures developed more cells with $G_1$-like DNA content but less $G_2-M$ cells relative to exponential-phase cultures. The median value of the RNA histogram for exponential cultures fell around channel 100 whereas the corresponding values for spheroid and plateau cultures occurred around channels 80 and 60 respectively. Clearly, there was a shift of the entire RNA distributions of spheroid and plateau cultures towards lower values relative to the exponential cells. This suggested that subpopulations of cells with low DNA and RNA (i.e., Q-cells) existed in both unelutriated spheroid and plateau cultures (Luk *et al.*, 1985; Luk *et al.*, 1986; Bauer *et al.*, 1982; Darzynkiewicz *et al.*, 1981; Traganos *et al.*, 1977). By using the histogram substraction program, the percentage of Q-cells in the latter cultures could be estimated. Essentially, this process involved comparing the RNA profiles of either the spheroids or the plateau cultures respectively with that of the asynchronously dividing exponential cells. The results yielded an estimate of 55% Q-cells for plateau and between 33% to 57% for spheroids of various sizes (Table I).

**Table II** Percentage of A431 cells in various cell-cycle compartments as determined by AO-staining

| *Type of culture* | **Geometric diameter (μm)* | $G_1$ | *S* | $G_2-M$ | *Q-cells* (%) |
|---|---|---|---|---|---|
| Exponential | – | 43 | 34 | 23 | – |
| | – | 41 | 35 | 24 | – |
| Fed Plateau | – | 65 | 26 | 9 | 55 |
| Spheroids | 462±8 | 50 | 36 | 14 | 33 |
| | 902±19 | 61 | 30 | 9 | 40 |
| | 1337±41 | 57 | 35 | 8 | 57 |

*Mean±s.e. of 50 spheroids.

In order to isolate the Q-cells, spheroids of ~900 μm diameter were dissociated and elutriated. Figure 4 depicts representative Coulter volume profiles of dissociated spheroid cells before and after elutriation. Unseparated cells typically gave a bimodal distribution encompassing a broad range of cell volumes from ~800 μm$^3$ to 4800 μm$^3$, indicating their heterogeneity in size (or volume). Elutriation, however, provided relatively homogeneous subpopulations of increasing size. Three of these subpopulations, labelled consecutively as A to C in their order of recovery (median volumes ~1380, 1790, 2100 μm$^3$) are displayed for comparison.

The DNA and RNA histograms from FCM analysis of the elutriated fractions were different (Figure 5). The DNA histogram analysis shows predominantly $G_1$-like DNA content in fractions A and B which was quite different from fraction C which contained a much greater proportion of S phase cells. The RNA distribution of fraction A, however, was observed at relatively lower values (channel numbers) than that of fraction B, indicating that although these two fractions contained cells with $G_1$-like DNA content they

**Figure 4** Representative cell volume profiles of dissociated A431 spheroids. Top panel shows the profile obtained from unelutriated spheroids. Bottom panel shows the 3 fractions recovered after elutriation ('A', 'B' and 'C') with approximate median volumes of 1380, 1790 and 2100 μm$^3$, respectively.

differed in their RNA content and possibly proliferative status (i.e., fraction A = Q-cells; fraction B = P-cells).

Continuous labelling of cells in spheroids with $^3$H-thymidine showed that only ~20% of the cells in fraction A were labelled after 48 h, confirming the quiescent nature of these cells. In contrast, ~78% of the cells in fraction B were labelled, indicating that they were predominantly proliferating cells. The control, unelutriated spheroid fraction comprised ~51% labelled cells (Table III). Table III also shows the plating efficiencies of these fractions which were not significantly different.

Figure 6 shows the Coulter volume distribution of trypsinized exponential-phase cells following centrifugal elutriation. Unseparated exponential cells ranged in volume from ~500 μm$^3$ to 4800 μm$^3$ (median volume ~2500 μm$^3$). Elutriated cells formed a homogeneous distribution with a

**Table III** Continuous labelling index and plating efficiency of elutriated A431 spheroid cells

| *Fraction* | **Labelling index* (%) | **Plating efficiency* (%) |
|---|---|---|
| Unseparated control | 54.0±3.1 | 30.7±2.2 |
| Separated Q | 19.8±3.6 | 33.7±2.0 |
| Separated $PG_1$ | 78.3±3.5 | 35.0±1.8 |

*Mean±s.e. of 3 experiments and in each experiment at least 400 cells were scored.

**Figure 5** Green fluorescence and red fluorescence histograms obtained by AO-staining FCM analysis of the various fractions recovered after elutriation of the A431 spheroids as described in **Figure 4**. Percentage of cells in various cell-cycle compartments: Unelutriated ($G_1=51, S=35, G_2-M=14$), A ($G_1=93.8, S=6.1, G_2-M=1$), B ($G_1=82, S=18, G_2-M=0$) and C ($G_1=52, S=47, G_2-M=1$). Note that another control comprised of exponential phase A431 cells (not shown here but see **Figure 3** for typical profiles of exponential phase cells) was routinely included for comparison with the unelutriated, spheroid control in these experiments.

**Figure 6** Representative cell volume profiles of A431 exponential cells. Top panel shows the profile before elutriation. Bottom panel shows the profile after elutriation with an approximate median volume of 1920 μm³.

small median volume (~1920 μm³). FCM analysis revealed that the separated cells had a DNA distribution compatible with cells with $G_1$-like DNA content but with a narrower range of RNA values relative to unseparated cells (Figure 7).

The radiation survival curves obtained with the Q-cells isolated from spheroids by elutriation demonstrated that they were more radiosensitive than the P-cells while the latter were quite similar in sensitivity to the exponential cells (Figure 8). It should be noted that all three fractions had $G_1$-like DNA content as determined by FCM analysis. The survival curve of Q-cells was steeper than that for P-cells and exponential cells. The various radiobiological parameters ($D_0$, $D_q$) are summarized in Table IV.

**Table IV** Radiobiological parameters of the various elutriated fractions

| *Cells* | *$D_0$(Gy) | *$D_q$(Gy) |
|---|---|---|
| $EG_1$ | 1.5±0.1 | 2.6 |
| $PG_1$ | 1.3±0.1 | 2.8 |
| Q | 0.9±0.1 | 2.6 |

$EG_1$: Exponential $G_1$ monolayer cells; $PG_1$: Proliferating $G_1$ spheroid cells; Q: Quiescent spheroid cells; *Mean±s.d. of 5 experiments.

### Discussion

Tumour heterogeneity may arise because of variations in the microenvironmental conditions such as pH, nutrition, oxygen status or osmolarity. Non-proliferating tumour cells arrested in $G_1$ (Denekamp, 1970; Pallavicini *et al.*, 1979) or in S and $G_2-M$ have been observed (Pallavicini *et al.*, 1979; Epinova & Terskitch, 1969). Using centrifugal elutriation, a relatively non-perturbing technique (Keng *et al.*, 1980), we have been able to isolate several subpopulations of cells of different sizes from the A431 human squamous carcinoma spheroids. Characterization of the elutriated subpopulations with FCM analysis and autoradiography demonstrated that the small

**Figure 7** Green fluorescence and red fluorescence histograms obtained by AO-staining FCM analysis of the $G_1$ fraction recovered after elutriation of the exponential cells described in **Figure 4**. Percentage of cells in various cell-cycle compartments: exponential, unelutriated ($G_1 = 45, S = 33, G_2 - M = 22$) and exponential, elutriated, $G_1$ ($G_1 = 95, S = 5, G_2 - M = 0$).

**Figure 8** Survival curves of A431 cells which were isolated and then irradiated with various doses. Note the following: fractions Q and $PG_1$ are equivalent to fractions A and B respectively from **Figures 4** and **5**, that is, they were derived from spheroids. Fraction $EG_1$, however, represents the elutriated, exponential monolayer cells isolated and characterized as described in **Figures 6** and **7**. Each group comprised 5 experiments; error bars refer to 1 s.e.

cell volume fractions recovered after elutriation were considerably enriched for Q-cells compared to control, unelutriated spheroid cells. This study therefore provided direct evidence of the presence of Q-cells in these human multicellular tumour spheroids. In addition, the recovered Q-cells were clonogenic, had $G_1$-like DNA content and were more radiosensitive than their proliferating counterparts, P-cells, derived under similar experimental conditions.

The 2-parameter AO-staining technique of Darzynkiewicz *et al.* (1981) and Traganos *et al.* (1977) formed the basis for the determination of the DNA and RNA distribution and content of the P- and Q-cells by FCM analysis. The clonogenicity of the isolated Q-cells showed that we were recovering real, intact cells and not merely cell nuclei or cytoplasmic fragments which might have DNA and RNA content resembling that of the former but would not have formed colonies after incubation. Evidence supporting the validity of the AO-staining technique for the determination of RNA content was provided by the finding that the mean red fluorescence of AO-stained A431 cells was linearly related to RNA content (unpublished) as was reported previously for HeLa and CHO cells (Bauer & Dethlefsen, 1981), and which we have confirmed for other cell lines. By making a similar assumption, we were able to estimate the number of Q-cells in this study from the red fluorescence obtained after AO-staining. Further confirmation of the quiescent status of the Q-cells was obtained from continuous $^3$H-thymidine labeling studies after a period equivalent to about one and a half cell-cycle times for the A431 cells.

It has been previously suggested by *in vitro* studies that Q-cells were less clonogenic than P-cells (Bauer *et al.*, 1982; Sigdestad & Grdina, 1981). This was speculated to support the thesis that Q-cells formed an intermediate compartment between P- and dead cells. In this study, there was no significant differences in clonogenicity between these cells. This observation agreed with recent mouse EMT6 mammary tumour data (Luk *et al.*, 1985, 1986). It should be noted, however, that the differences in clonogenicity reported among P- and Q-cells from the various studies might be reflecting different modes of induction of Q-cells from those studies, for example, by nutrient-deprivation, catabolite accumulation, hypoxia, etc. and/or the ability of the Q-cell population in different cell lines to remain clonogenic under various conditions.

Q-cells isolated from human tumor spheroids in this study have been found to be more radiosensitive than P-cells from the same spheroids, in agreement with previous results for EMT6 spheroids (Luk, 1986). However, this does not necessarily indicate that these cells are not important to radiation therapy. This is because Q-cells may well turn out to be more competent in repairing radiation damage than P-cells. A comparison of Q-cell radiosensitivity with exponential phase $G_1$-cells showed that the latter were also more radioresistant. This study supports the concept that subpopulations of cells with varying degrees of quiescence exhibit different sensitivities to different therapeutic modalities and must be considered further relative to recruitment and repair potentials in order to understand overall responses to therapy.

We thank C. Edler, B. King and S. Bui for technical support. Part of this work was done in the Cell Separation Facility, Cancer Center. This work was supported by grants number CA 11198, CA 11051, CA 37618 and CA 20329 from the National Cancer Institute of the National Institutes of Health.

### References

BAUER, K.D. & DETHLEFSEN, L.A. (1981). Control of cellular proliferation of HeLa-S3 suspension cultures. Characterization of cultures utilizing acridine orange staining procedures. *J. Cell. Physiol.*, **108**, 99.

BAUER, K.D., KENG, P.C. & SUTHERLAND, R.M. (1982). Isolation of quiescent cells from multicell spheroids using centrifugal elutriation. *Cancer Res.*, **42**, 72.

CLARKSON, B. (1974). The survival value of the dormant state in neoplastic and normal cell populations. In *Control of Proliferation in Animal Cells*, Clarkson B.D. & Baserga, R. (eds) p. 945. Cold Spring Harbor, Cold Spring Harbor Laboratory. NY.

DARZYNKIEWICZ, Z., TRAGANOS, R. & MELAMED, M.R. (1981). New cell cycle compartments identified by multiparameter flow cytometry. *Cytometry*, **1**, 98.

DENEKAMP, J. (1970). The cellular proliferation kinetics of animal tumors. *Cancer Res.*, **30**, 393.

DEXTER, D.L. & CALABRESI, P. (1982). Intraneoplastic diversity. *Biochem. Biophys. Acta.*, **695**, 97.

DETHLEFSEN, L.A. (1979). In quest of the quaint quiescent cells. In *Radiation Biology in Cancer Research*, Meyn, R.E. & Withers H.R. (eds) p.415. Raven Press: New York.

EPINOVA, O.I. & TERSKITCH, V.V. (1969). On the resting periods in the cell life cycle. *Cell Tissue Kinet.*, **2**, 75.

FREYER, J.P. & SCHOR, P.L. (1985). Radiation survival of quiescent cells from tumor spheroids. *Abstracts of Papers for the 33rd Annual Meeting of the Radiation Research Society*, Los Angeles, Ca.

FRIED, J. & MANDEL, M. (1979). Multiuser system for analysis of data from flow cytometry. *Comput. Programs Biomed.*, **10**, 218.

GIARD, D.J., AARONSON, S.A., TODARO, G.J. & 4 others (1973). *In vitro* cultivation of human tumors: Establishment of cell lines derived from a series of solid tumors. *J. Natl Cancer Inst.*, **51**, 1417.

HEPPNER, G.H. & MILLER, B.E. (1983). Tumor heterogeneity: Biological implications and therapeutic consequences. *Cancer Metastasis Rev.*, **2**, 5.

KALLMAN, R.F., COMBS, C.A., FRANKS, A.J. *et al.* (1979). Evidence for the recruitment of noncycling clonogenic tumor cells. In *Radiation Biology in Cancer Research*, Meyn, R.E. & Withers H.R. (eds) p.397. Raven Press: New York.

KENG, P.C., LI, C.K. & WHEELER, K.T. (1980). Synchronisation of 9L rat brain tumor cells by centrifugal elutriation. *Cell Biophys.*, **2**, 191.

LUK, C., KENG, P.C.K. & SUTHERLAND, R.M. (1985). Regrowth and radiation sensitivity of quiescent cells isolated from EMT6/Ro-fed plateau monolayers. *Cancer Res.*, **45**, 1020.

LUK, C., KENG, P.C.K. & SUTHERLAND, R.M. (1986). Radiation response of proliferating and quiescent subpopulations isolated from multicellular spheroids. *Br. J. Cancer*, **54**, 25.

MILLER, F.R. (1982). Intratumor heterogeneity. *Cancer Metastasis Rev.*, **1**, 319.

OWENS, A., COFFREY, D.S. & BAYLIN, S.B. (eds.) (1982). *Tumor Cell Heterogeneity, Origins and Implications*, Academic Press Inc.: New York.

PALLAVICINI, M.G., LALLANDE, M.E., MILLER, R.G. & HILL, R.P. (1979). Cell cycle distribution of chronically hypoxic cells and determination of clonogenic potential of cells accumulated in $G_2$ and M phases after irradiation of a solid tumor *in vivo*. *Cancer Res.*, **39**, 1891.

POTMESIL, M. & GOLDFEDER, A. (1980). Cell kinetics of irradiated experimental tumors: Cell transition from the nonproliferating to the proliferating pool. *Cell Tissue Kinet.*, **13**, 563.

SIGDESTAD, C.P. & GRDINA, D.J. (1981). Density centrifugation of murine fibrosarcoma cells following *in situ* labeling with tritiated thymidine. *Cell Tissue Kinet.*, **14**, 589.

SUTHERLAND, R.M. (1974). Selective chemotherapy of noncycling cells in an *in vitro* tumor model. *Cancer Res.*, **34**, 3501.

SUTHERLAND, R.M. & DURAND, R.E. (1976). Radiation response of multicell spheroids – an *in vitro* tumor model. *Current Top. Radiat. Res.*, **11**, 87.

SUTHERLAND, R.M., EDDY, H.A., BAREHAM, B. *et al.* (1979). Resistance to Adriamycin in multicellular spheroids. *Int. J. Radiat. Oncol. Phys.*, **5**, 1225.

SUTHERLAND, R.M., McCREDIE, J.A. & INCH, W.R. (1971). Growth of multicell spheroids in tissue culture as a model of nodular carcinomas. *J. Natl Cancer Inst.*, **46**, 113.

TRAGANOS, F., DARZYNKIEWICZ, Z., SHARPLESS, T. & MELAMED, M.R. (1977). Simultaneous staining of ribonucleic and deoxyribonucleic acids in unfixed cells using acridine orange in a flow cytofluorometric system. *J. Histochem. Cytochem.*, **25**, 46.

WALLEN, C.A., HIGASHIKUBO, R. & DETHLEFSEN, L.A. (1984). Murine mammary tumor cells *in vitro*. II. Recruitment of quiescent cells. *Cell. Tissue Kinet.*, **17**, 79.

WOODRUFF, M.F.A. (1983). Cellular heterogeneity in tumors. *Br. J. Cancer*, **47**, 589.

YUHAS, J.M., TARLETON, A.E. & HARMAN, J.G. (1978). *In vitro* analysis of the radiation response of multicellular tumor spheroids exposed to chemotherapeutic agents *in vitro* or *in vivo*. *Cancer Res.*, **38**, 3595.